# The Role of the 3' Untranslated Region in the Post-Transcriptional Regulation of KLF6 Gene Expression in Hepatocellular Carcinoma

**DOI:** 10.3390/cancers6010028

**Published:** 2013-12-19

**Authors:** Thoria Diab, Naima Hanoun, Christophe Bureau, Camille Christol, Louis Buscail, Pierre Cordelier, Jérôme Torrisani

**Affiliations:** 1 INSERM UMR 1037, Toulouse 31432, France; E-Mails: thoria.diab@inserm.fr (T.D.); naima.hanoun@inserm.fr (N.H.); bureau.c@chu-toulouse.fr (C.B.); christol.c@chu-toulouse.fr (C.C.); buscail.l@chu-toulouse.fr (L.B.); jerome.torrisani@inserm.fr (J.T.); 2 Paul Sabatier University, Toulouse 31000, France; 3 Department of Hepatology, Toulouse University Hospital Centre, Toulouse 31409, France; 4 Department of Gastroenterology, Toulouse University Hospital Centre, Toulouse 31409, France

**Keywords:** hepatocellular carcinoma, KLF factors, KLF6, untranslated region, gene expression

## Abstract

KLF6 is ubiquitously expressed in human tissues and regulates many pathways such as differentiation, development, cellular proliferation, growth-related signal transduction, and apoptosis. We previously demonstrated that KLF6 expression is altered during liver carcinogenesis. More importantly, KLF6 invalidation results in cell cycle progression inhibition and apoptosis of liver cancer cells. On the other hand, enforced expression of KLF6 variant 2 (SV2) induces cancer cell death by apoptosis. Thus, we and others demonstrated that KLF6 and its splicing variants play a critical role in liver cancer. However, little is known on the mechanisms governing KLF6 expression in HCC. In the present work, we asked whether the 3' untranslated region (3'UTR) of the KLF6 mRNA may be responsible for regulation of KLF6 expression in HCC. We found that KLF6 mRNA stability was altered in liver-derived cell lines as compared to cervical cancer-derived cell lines and human embryonic fibroblasts. Interestingly, KLF6 mRNA was highly unstable in liver cancer-derived cell lines as compared to normal hepatocytes. We next cloned the KLF6 mRNA 3'UTR into luciferase-expressing vectors and found that gene expression and activity were strongly impaired in all liver-derived cell lines tested. In addition, we found that most the KLF6 3'UTR destabilisation activity resides between nt 1,835 and nt 2,615 of the KLF6 gene. Taken together, we provide the first steps towards better understanding of the regulation of KLF6 expression in HCC. Further work is needed to identify the factors that bind to KLF6 3'UTR to regulate its expression in liver cancer-derived cell lines.

## 1. Introduction

Krüppel-like factor 6 (KLF6, Zf9, COPEB) belongs to the family of Sp1-like/KLF transcription factors that contain three highly conserved Cys2-His2 type zinc fingers located in the *C*-terminus and a proline and serine rich NH_2_ terminal activation domain [1]. The KLF6 gene is located on chromosome arm 10p and has four exons separated by three introns and encodes for a protein of 283 amino acids [2,3]. KLF6 is ubiquitously expressed in human tissues and regulates many pathways such as differentiation, development, cellular proliferation, growth-related signal transduction, and apoptosis [1,4]. KLF6 may also play a more generalized role in tumorigenenesis as a tumor suppressor gene that is inactivated in a number of human cancers by loss of heterozygosity (LOH) [5], somatic mutation, promoter hypermethylation [6] decreased expression [7] and increased alternative splicing into a dominant negative oncogenic splice variant, KLF6-SV1 [8]. KLF6 inactivation has been implicated in a number of human cancers, including colorectal [9] non-small cell lung [10] gastric [11], astrocytic glioma [12], and nasopharyngeal [13]. 

We and other have demonstrated that KLF6 is also downregulated during liver carcinogenesis [4,7,14,15] and this downregulation may protect cancer cells from apoptosis [15]. Interestingly, KLF6 modulation in cancer cells potentially alters cell cycle progression [15]. In addition, we found that the SV2 variant of KLF6 is downregulated in hepatocellular carcinoma (HCC) that results in inhibition of cell proliferation and induction of apoptosis [16]. Thus, KLF6 and its variants may play a pivotal role during HCC [17]. 

The mechanisms responsible for KLF6 decreased expression in HCC are still poorly understood. Indeed, no somatic mutations of KLF6 are observed in patients with dysplastic nodules or with HCC, and allelic loss is a very rare event [18]. In methylation analysis, KLF6 gene was found fully methylated in only one (out of 85) HCC tissue [18]. Taken together, genetic and epigenetic alterations may play a minor role in the regulation of KLF6 expression in HCC. Modification of transcript stability allows gene expression to be rapidly controlled without altering transcription and translation rates. This mechanism has been found to be critically involved in vital processes such as cell growth and differentiation, as well as adaptation to external stimuli [19,20]. The 3' untranslated region (3'UTR), situated downstream of the protein coding sequence, has been found to be involved in numerous regulatory processes that are critical in determining the fate of mRNAs including transcript cleavage, stability and polyadenylation, translation and mRNA localization [21] In contrast to the promoter region, motifs in the 3'UTR are primarily conserved, which is consistent with the 3'UTR acting to regulate gene expression at the post-transcriptional level [22]. The 3'UTR serves as a binding site for numerous regulatory proteins as well as for microRNAs. In the present work, we asked whether KLF6 3'UTR may be responsible for down regulation of KLF6 mRNA in HCC, and therefore may contribute to cell cycle alteration of liver cancer cells. 

## 2. Experimental

*Cell lines*: Immortalized Human Hepatocytes (IHH), HepG2, Hep3B (hepatoma cell lines) and HeLa-S3 (immortal cell line derived from cervical cancer cells) were cultured in DMEM (Dulbecco modified Eagle) medium glucose containing 4.5 g/L of glucose and supplemented with 10% fetal calf serum (Invitrogen, Saint Aubin, France), antibiotic (1%) and L-glutamine (2 mM, Invitrogen). All cell lines were grown in 5% CO_2_ at 37 °C in the presence of Plasmocin (Cayla Invivogen, Toulouse, France) to prevent mycoplasma contamination.

*Actinomycin treatment*: Two million cells were cultured in 100-mm culture dish for 24 h. Cells were then treated with actinomycin D (ActD, 5 µg/mL) for 0, 1, 2 and 4 h. 

*RNA extraction, reverse transcription and quantitative PCR*: Total RNA was extracted using TRIZOL reagent (Invitrogen), RNAs were quantitated by NanoDrop 2000. To eliminate amplification of reporter plasmid, RNA (1 µg) was treated with DNase (Invitrogen). Five µg of RNA was reverse transcribed using random primers and RevertAid reverse transcriptase (Fermentas, Illkirch, France). Quantitative real-time PCR was performed using SsoFast EvaGreen Super Mix (BioRad, Marnes-la-Coquette, France) and the primers listed in Table 1 using a StepOnePlus apparatus. Experiments were performed in duplicate. Gene expression was normalized using glyceraldehyde-3-phosphate dehydrogenase (GAPDH) as house-keeping gene. All results were analyzed using the stepone 2.2 software and the mRNA expression was calculated according to the formula: 2exp^−(Ct target gene − Ct reference gene)^.

### 2.1. Construction of Plasmids

*KLF6 3'UTR*: Full-length KLF6 3'UTR was PCR-amplified from Bacterial Artificial Chromosome (BAC, BACPAC Resource Center (BPRC) from the Children’s Hospital Oakland Research Institute, Oakland, CA, USA). PCR was performed using Phusion PCR Master Mix (Thermo Scientific, Waltham, MA, USA) using KLF6 3'UTR F2 GW and 3'UTR R2 GW primers (Table 1) according to the following steps: denaturation for 10 min at 98 °C, followed by 35 cycles of amplification (15 s of denaturation at 98 °C, 15 s of annealing at 57 °C, 1 min of extension at 68 °C) and a final extension at 68 °C for 10 min. PCR products and psiCHECK2 plasmid (Promega, Lyon, France) were double-digested with XhoI and NotI restriction enzymes (Fermentas). Digestion products were purified using NucleoSpin® extract II kit (Macherey-Nagel, Hoerdt, France) and visualized by ethidium bromide before ligation using T4 DNA Ligase (Invitrogen) overnight at 16 °C to give psiCHECK2-Gateway KLF6 3'UTR FL. In this construct, firefly luciferase expression driven by HSV-TK promoter serves as a reference, thus avoiding co-transfection of control plasmid. All plasmids were verified by automated sequencing (Millegen, Toulouse, France).

**Table 1:** Primer sequences used for PCR amplification.

Primer	Sequence
KLF6 F	CGG ACG CAC ACA GGA GAA AA
KLF6 R	CGG TGT GCT TTC GGA AGT G
UTR1F GW	GGGG ACA AGT TTG ATC AAA AAA GCA GGC TGG GAG CAG AGA GGT GGA TCC T
UTR1R GW	GGGG AC CAC TTT GTA CAA GAA AGC TGG GTT ACA CAG CTT ATA CAA TGG GTT ACA AAT G
UTR2F GW	GGGG ACA AGT TTG TAC AAA AAA GCA GGC TGT GTC AAG TAG CTT GTT TTA CAC GCT AC
UTR2R GW	GGGG AC CAC TTT GTA CAA GAA AGC TGG GTA AGG TCT ATA TGA AAG TCT CAA GGT GGC
UTR3F GW	GGGG ACA AGT TTG TAC AAA AAA GCA GGC TGA CTG TCA GTG TTA AAA TGG AAA ACA GG
UTR3R GW	GGGG AC CAC TTT GTA CAA GAA AGC TGG GTA TAA AGC AAA GAG CCA CAC CCA C
UTR4F GW	GGGG ACA AGT TTG TAC AAA AAA GCA GGC TAA TTG GCA TAC CAC GGC GTG
UTR4R GW	GGGG AC CAC TTT GTA CAA GAA AGC TGG GTT GGC AGT GAT GTC ATC TTT TAT TTT CTG

Segments of KLF6 3'UTR (3'UTR1 1,114–1,834, 720 bp, 3'UTR2 1,835–2,615, 780 bp, 3'UTR3 2,616–3,486, 870 bp, and 3'UTR4 3,486–4,786, 1,300 bp) were PCR-amplified from HepG2 cDNA using UTR2, UTR3 and UTR4 primers (Table 1). The PCR products were gel-purified and cloned into pGEMT Easy vector (Promega). The resulting plasmids were digested using EcoR1 and verified by agarose gel electrophoresis. A set of Gateway primers (Invitrogen) containing attB (GGGG ACA AGT TTG TAC) and attB2 (GGGG AC CAC TTT GTA) sequences (Table 1) was designed for PCR amplification of KLF6 UTR segments from pGEMT KLF6 3'UTR1, 2, 3 and 4 using Phusion PCR Master Mix for 35 cycles with a Tm of 55 °C. The purified DNAs were gateway cloned into pDONOR^TM^221 using BP reaction to give pDONR KLF6 UTR1, 2, 3 and 4. The pDONOR^TM^221 vector was LR crossed with psi-Check2 to give psi-Check2 KLF6 UTR1, 2, 3 and 4. All recombinant plasmids were verified by automated sequencing (Millegen).

*Bacterial transformation and plasmid amplification*: The recombinant psiCHECK2 GW KLF6 3'UTR DNAs were transformed into Top10 competent bacteria (Invitrogen). One colony was inoculated into 2 mL LB medium containing 100 µg/mL ampicillin and shaken overnight at 37 °C. DNA was purified by using NucleoSpin® Plasmid (Macherey-Nagel) kit. 500 µL of the overnight culture were inoculated into 250 mL LB medium with 100 µg/mL ampicillin and shaken at 37 °C over night. DNA was purified by using GenElute^TM^ HP Plasmid Maxi prep Kit (Sigma, St Louis, MO, USA). 

*Transfection*: 2 × 10^4^ IHH, HepG2 and Hep3B cells were retrotransfected in 48-well dishes using 0.25 µg of plasmid and 12.5 µL of LyoVec^TM^ transfection reagent (Invivogen). HeLa-S3 cells (2 × 10^4^ cells) were plated in 48-well dishes 24 h before transfection. Cells were transiently transfected with 0.5 µg of plasmid using PEI (Sigma), using a 3:1 ratio of PEI/DNA.

*Reporter Assay*: Luciferase reporter assays were performed using the dual-luciferase Reporter Assay system (Promega). Cells were harvested 48 h following transfection. The culture medium was removed, and cells were washed with phosphate-buffered saline (PBS). Passive lysis buffer (1×) was added to each well. Cells were frozen at −20 °C for 15 min, and lysates were prepared using the dual-luciferase Reporter Assay system (Promega). Luciferase activity in was determined 20 μL of lysate using a luminometer (Luminoskan Ascent, Thermo). Renilla luciferase activity was normalized to firefly activity. Both luciferase measurements were done in duplicate.

### 2.2. Statistics and Software

Bioinformatic analyses of KLF6 3'UTR were performed at [23]. The averages, standard deviations and student t-test were calculated using GraphPAD Prism software.

## 3. Results

### 3.1. Bioinformatic Analysis of KLF6 3'UTR

KLF6 is ubiquitously expressed in human tissues and cell lines. However, we and others demonstrated that its expression is altered during HCC and during hepatocellular carcinogenesis [4,14,24]. Previous reports demonstrated that genetic and epigenetic changes do not account for the downregulation of KLF6 expression in this disease [22]. Consequently, we asked whether KLF6 3' UTR may regulate KLF6 expression during liver carcinogenesis.

**Figure 1:**
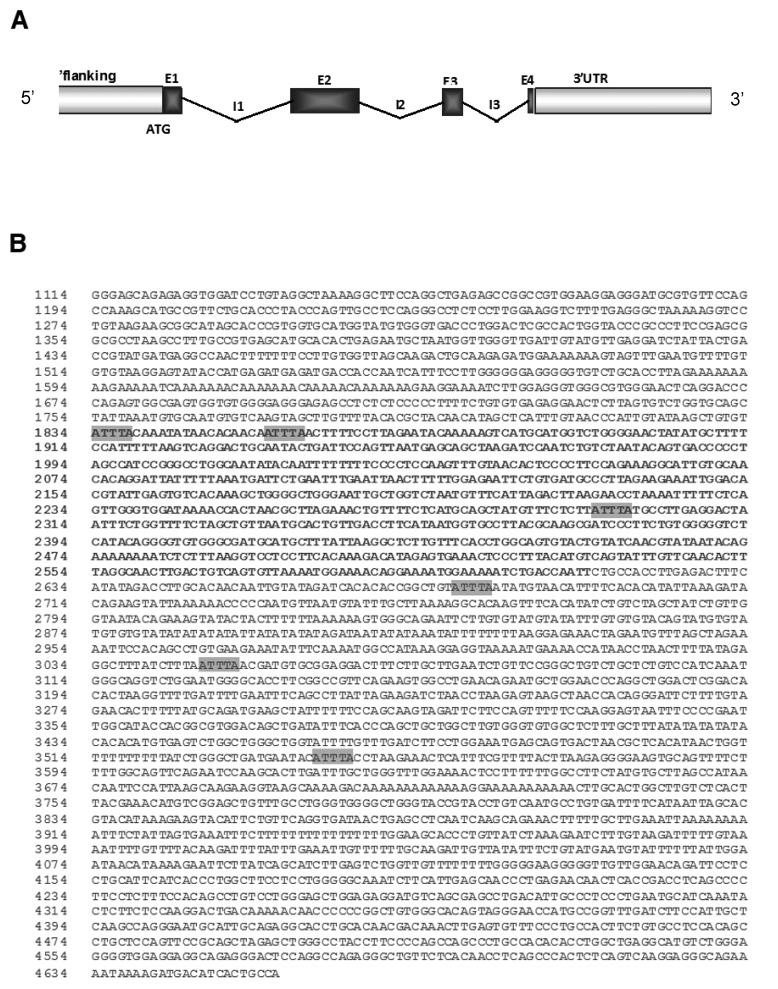
Organization of the KLF6 gene. (**A**) Schematic representation of the KLF6 gene, including untranslated regions (UTR), introns (I) and Exons (E); (**B**) Bioinformatic analysis of KLF6 UTR for AU-rich Elements (highlighted in grey). KLF6 UTR segment #2 is highlighted in bold. AU-rich elements were identified using [23].

The 3'UTR of the human *KLF6* gene is almost 4 kb long (Figure 1A) while the mean size of 3'UTRs of human genes is around 800 nucleotides [16]. As shown in Figure 1B, bioinformatic analysis of this region revealed the presence of six AU-rich Elements (ARE) highlighted in grey composed of AUUUA pentamers. Among them, three are found in the UTR2 region that is designated in bold. This preliminary analysis suggests that ARE located in KLF6 3'-UTR may play a functional role in the post-transcriptional regulation of KLF6 expression. 

### 3.2. Determination of KLF6 mRNA Half-Life

**Figure 2:**
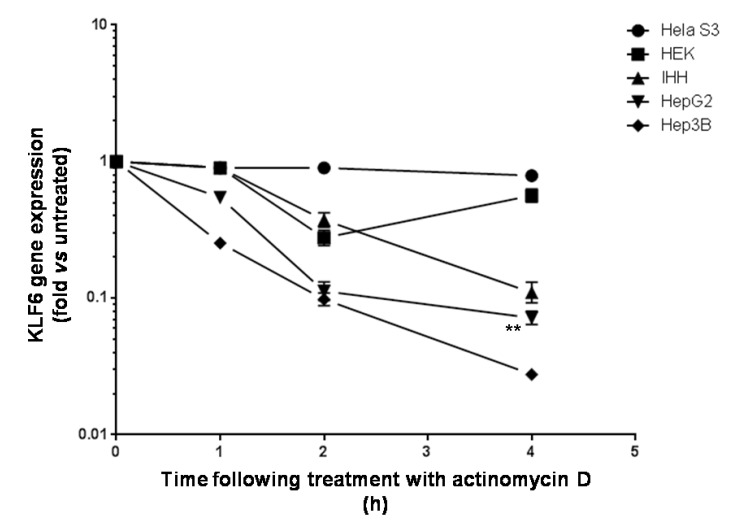
Determination of KLF6 mRNA half life in hepatic and non-hepatic cell lines. Hela, HEK, IHH, HepG2 and Hep3B cells were treated with Actiomycin D as described in Experimental Section. KLF6 mRNA expression was quantified by qRT-PCR at the times indicated. Results are mean ± S.D. of three independent experiments. **: *p* < 0.01.

We next determined the stability of KLF6 mRNA *in vitro*. As shown in Figure 2, KLF6 mRNA decreased within 45 min to 4 h in the presence of the transcription inhibitor actinomycin D and was reduced by 95% after 4 h of treatment. Interestingly, KLF6 mRNA half life greatly differed in the cell lines tested. We found that KLF6 mRNA is fairly stable in HeLa-S3 (t_1/2_ = 2.34 h) and HEK-293T (t_1/2_ = 1.4 h) cells. However, KLF6 mRNA half life greatly decreases in HCC-derived cell lines (t_1/2_ = 0.68 h for Hep3B, t_1/2_ = 0.82 h for HepG2) as compared to normal human hepatocytes (t_1/2_ = 1.05 h for IHH). KLF6 mRNA decay was significantly faster in human liver cancer cell lines (Hep3B and HepG2) as compared to immortalized human hepatocytes (IHH) (student t test, *p* = 0.008).

### 3.3. KLF6 3'UTR Is a Potent Regulator of Gene Expression

**Figure 3:**
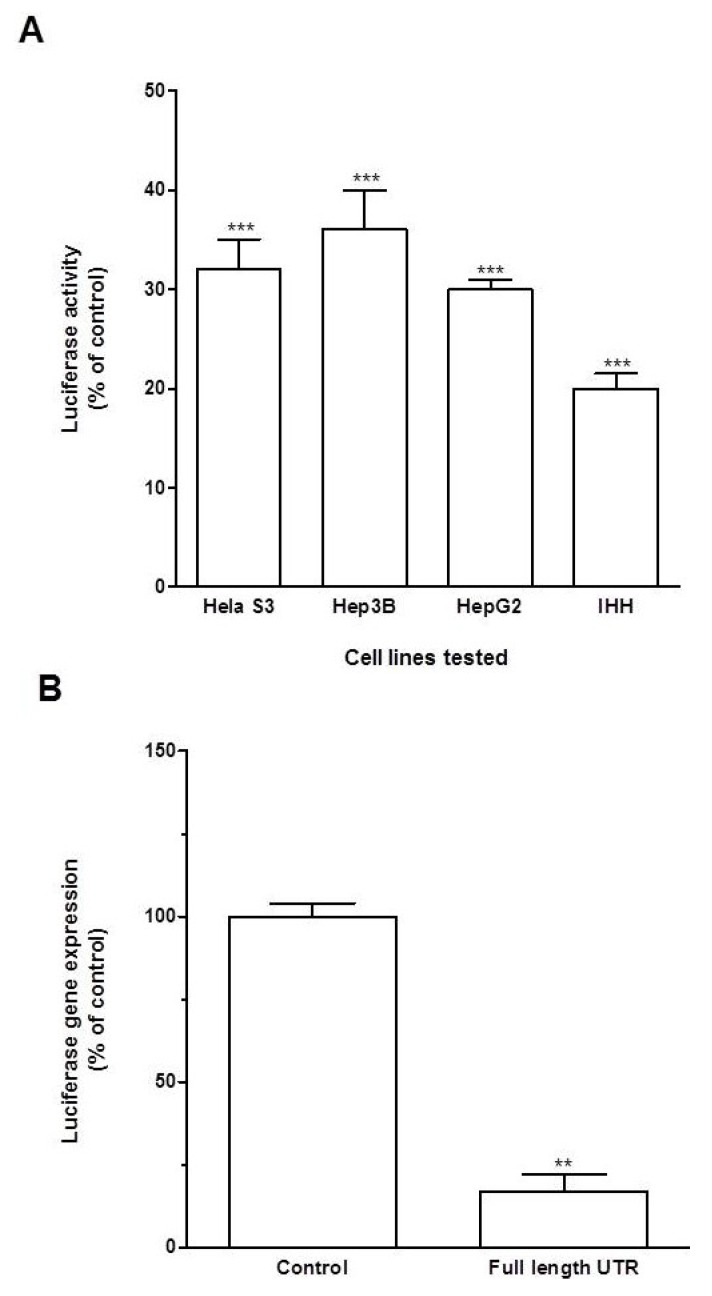
Effect of KLF6 UTR on gene expression. Hela, IHH, HepG2 and Hep3B cells were transfected for 48 h with plasmid containing KLF6 UTR. Control cells were transfected with psiCHECK2 plasmid. (**A**) Luciferase activity was determined as described in Experimental Section. Results are mean ± S.D. of three independent experiments. ***: *p* < 0.001; (**B**) Luciferase gene expression was measured by qRTPCR in Hela cells following plasmid transfection as described in Experimental Section. Results are mean ± S.D. of three independent experiments. **: *p* < 0.01.

In order to assess the role of KLF6 3'UTR in the regulation of KLF6 stability, HeLa S3, IHH, HepG2 and Hep3B cells were transiently transfected with luciferase-encoding vectors (psi-Check2) with the KLF6 3'UTR inserted downstream of the renilla luciferase coding region. Control cells were transfected with empty psiCHECK2 plasmid. 48 h after transfection, cells were lysed and luciferase activity was measured in total cellular extracts. As shown in Figure 3A, KLF6 3'UTR profoundly altered the expression of the luciferase reporter gene, as compared to control transfected cells. Luciferase activity was decreased by 70%–80% in all cell lines tested receiving the KLF6 UTR-expressing plasmid. 

We next asked whether KLF6 UTR inhibitory effect on luciferase activity was due to inhibition of mRNA expression or repression of translation. Consequently, we quantified renilla luciferase mRNA by qRTPCR. HeLa-S3 cells were transfected with psi-Check2 KLF6 3UTR FL and psi-Check2 empty vector as a control. Renilla luciferase expression was determined by RT-qPCR and normalized to firefly luciferase mRNA. The construct containing KLF6 3'UTR reduced luciferase mRNA by ~85% as compared to control (Figure 3B) iindicating that KLF6 3'UTR affects the expression of the reporter gene at the transcriptional level. Taken together, we demonstrate that KLF6 3'UTR negatively regulates mRNA stability to repress reporter gene expression.

**Figure 4:**
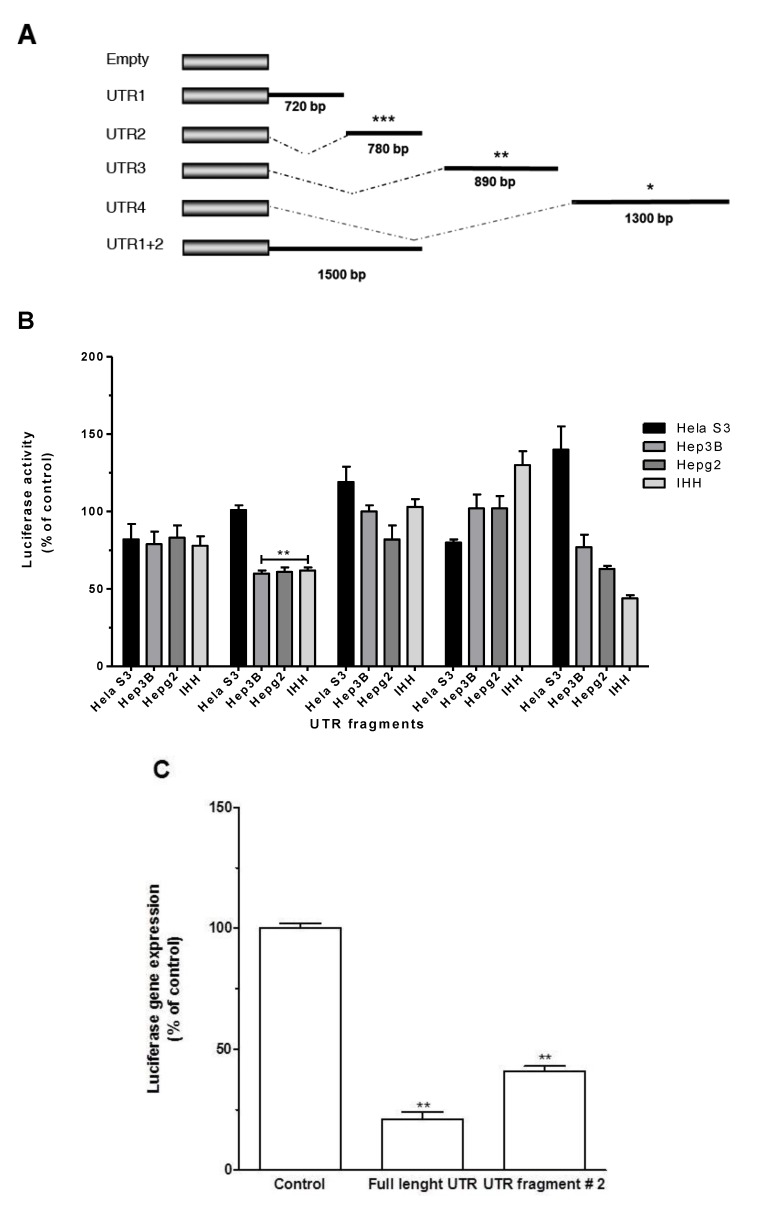
Molecular dissection of KLF6 UTR. (**A**) Representation of the psiCHECK2-derived constructs containing segment 1 (nt 1,114–1,834), 2 (nt 1,835–2,615), 3 (nt 2,616–3,506) or 4 (3,507–4,807) of the KLF6 3'UTR. Segment size is indicated in bold. Hela, IHH, HepG2 and Hep3B cells were transfected for 48 h with plasmid encoding for the indicated KLF6 UTR segments. Control cells were transfected with psiCHECK2 plasmid; (**B**) Luciferase activity was determined as described in Experimental Section. Results are mean ± S.D. of three independent experiments. **: *p* < 0.01; (**C**) Luciferase gene expression was measured by qRTPCR in Hela cells following plasmid transfection as described in Experimental Section. Results are mean ± S.D. of three independent experiments. **: *p* < 0.01.

As indicated by our bioinformatic analysis, five ARE recognition sequences are located in different regions of the KLF6 3'UTR. To better understand which region of KLF6 3'UTR is responsible for its destabilisation activity, we generated four segments of the KLF6 3'UTR (UTR1, UTR2, UTR3 and UTR4) as described in the Experimental section that were cloned into psiCHECK 2 (Figure 4A). The resulting plasmids were transfected into the cell lines tested as described in the Experimental section. Control cells were transfected with empty psiCHECK2 plasmid. As shown in Figure 4B, none of the constructs tested reduced luciferase activity in Hela S3 cells. On the other hand, KLF6 UTR segment 2 reduced the reporter gene activity by ~45% in hepatic cell lines, compared to other 3'UTR segments (Figure 4B). To address whether the decrease in the luciferase activity observed is due to mRNA degradation or blocking of protein translation, we quantified luciferase mRNA by RT-qPCR in transfected cells. As shown in Figure 4C, the construct containing KLF6 3'UTR segment 2 reduced luciferase mRNA by 50% as compared to control further indicating that KLF6 3'UTR affects the expression of the reporter gene at the post-transcriptional level.

## 4. Discussion

KLF6 is ubiquitously expressed in human tissues and regulates many pathways such as differentiation, development, cellular proliferation, growth-related signal transduction, and apoptosis [1,4]. We previously demonstrated that KLF6 is profoundly down regulated during liver carcinogenesis [5,7,14,15] and may protect cancer cells from apoptosis [15]. In addition, KLF6 splicing variant SV2 is down-regulated in hepatocellular carcinoma (HCC) and may act as an inhibitor of cell proliferation and as a promoter of cell death by apoptosis [16]. Taken together, KLF6 plays a pivotal role during HCC [17]. 

Little is known about the mechanisms governing KLF6 expression in HCC. To date, somatic mutations of KLF6 have not been detected in patients with dysplastic nodules or with HCC, and allelic loss is a very rare event ([18] and personal observations). It was proposed for decades that DNA methylation might be responsible for the stable maintenance of a particular gene expression pattern through mitotic cell division. Since then, ample evidence has been obtained to support this concept, and DNA methylation is now recognized to be a chief contributor to the stability of gene expression states. Specifically, DNA methylation establishes a silent chromatin state by collaborating with proteins that modify nucleosomes. In methylation analysis, only one HCC tissue showed methylated DNA without unmethylated DNA [18]. Taken together, genetic and epigenetic alterations may play a minor role in the regulation of KLF6 expression in HCC. 

Regulatory regions within the 3'UTR region can influence polyadenylation, translation efficiency, localization, and stability of the mRNA. Modification of transcript stability allows expression to be rapidly controlled without altering translation rates. Interestingly enough, we found that KLF6 UTR is almost 4 kb long, while the mean size of 3'UTRs of human genes is around 800 nucleotides [25]. This strongly suggests that KLF6 UTR may be responsible for the regulation of KLF6 mRNA expression. Accordingly, we measured KLF6 mRNA half life in cell lines including cells derived from normal or cancerous human liver. We found that KLF6 mRNA half life was greatly decreased in cells derived from HCC. We next cloned KLF6 UTR and demonstrated that reporter gene expression and activity were greatly decreased in plasmid containing this untranslated region. In addition, we narrowed the destabilisation activity of KLF6 UTR segment #2 (1,835–2,615). These segments specifically disrupted KLF6 mRNA integrity in hepatic-derived cell lines. We identified several AREs in KLF6 UTR segment #2. A number of RNA binding proteins that recognize AREs and regulate the translation and degradation of ARE-mRNAs have been identified. These proteins are regulated by signal transduction pathways, which are often misregulated in cancers. Interestingly, AUF1 and Hur, two major RNA binding proteins have been recently demonstrated to support HCC growth through the posttranslational regulation of two methionine adenosyltransferase [26]. Accordingly, we recently performed *in vitro* transcription assays to generate KLF6 UTR segment #2 to use it as bait in surface plasmon resonnance experiments. This strategy may lead to the identification of proteins, such as AUF1 and/or Hur, that bind to the KLF6 3'UTR. Once characterized, we will modulate the expression of the candidate proteins in cell lines and will study KLF6 expression. We will next correlate KLF6 and the candidate proteins expression in TMA of HCC. 

Beside AU-rich binding proteins, the 3'UTR of mRNA contains binding sites for microRNAs (miRNAs). By binding to specific sites within the 3'UTR, miRNAs can decrease gene expression of various mRNAs by either inhibiting translation or directly causing degradation of the transcript [27]. Recent work by Zhang *et al.* suggests that KLF6 is a direct target of miR-181a [28]. Interestingly, they demonstrate that miR-181a functions as a tumor promoter in gastric cancer by repressing the expression of KLF6. In another study, Tsai *et al.* found that microRNA-122 (miR-122), which accounts for 70% of the liver’s total miRNAs, targets KLF6 [24]. Interestingly, livers from miR-122 KO mice exhibited disruptions in a range of pathways, many of which closely resemble the disruptions found in human HCC. Importantly, the reexpression of miR-122a reduced disease manifestation and tumor incidence in miR-122a KO mice. These results strongly suggest that miR-122 miRNA may account for KLF6 reduced expression in normal liver. Interestingly, those two candidate miRNAs known to target KLF6 do not target the segment 2 of its 3'UTR described herein, adding another level of complexity in the regulation of KLF6 expression in liver cells. We are currently exploring whether miR-181a and miR-122 regulate KLF6 expression in liver-derived cancer cells. 

## 5. Conclusions

Taken together, we provide herein the first steps that hold great promise to better understand the regulation of KLF6 expression in HCC. Further work is needed to identify the miRNAs and/or the proteins that bind to KLF6 UTR to regulate its expression in liver cancer-derived cell lines, and therefore may contribute to cell cycle alteration of liver-derived cancer cells.

## Abbreviations

HCC: hepatocellular carcinoma
KLF: Kruppel-like factor
UTR: untranslated region

## References

[1] Black A.R., Black J.D., Azizkhan-Clifford J. (2001). Sp1 and krüppel-like factor family of transcription factors in cell growth regulation and cancer. J. Cell. Physiol..

[2] Benzeno S., Narla G., Allina J., Cheng G.Z., Reeves H.L., Banck M.S., Odin J.A., Diehl J.A., Germain D., Friedman S.L. (2004). Cyclin-dependent kinase inhibition by the KLF6 tumor suppressor protein through interaction with cyclin D1. Cancer Res..

[3] Rodríguez E., Aburjania N., Priedigkeit N.M., DiFeo A., Martignetti J.A. (2010). Nucleo-cytoplasmic localization domains regulate Krüppel-like factor 6 (KLF6) protein stability and tumor suppressor function. PLoS One.

[4] Bieker J.J. (2001). Krüppel-like factors: Three fingers in many pies. J. Biol. Chem..

[5] Kremer-Tal S., Reeves H.L., Narla G., Thung S.N., Schwartz M., Difeo A., Katz A., Bruix J., Bioulac-Sage P., Martignetti J.A. (2004). Frequent inactivation of the tumor suppressor Kruppel-like factor 6 (KLF6) in hepatocellular carcinoma. Hepatology.

[6] Yamashita K., Upadhyay S., Osada M., Hoque M.O., Xiao Y., Mori M., Sato F., Meltzer S.J., Sidransky D. (2002). Pharmacologic unmasking of epigenetically silenced tumor suppressor genes in esophageal squamous cell carcinoma. Cancer Cell.

[7] Kremer-Tal S., Narla G., Chen Y., Hod E., DiFeo A., Yea S., Lee J.-S., Schwartz M., Thung S.N., Fiel I.M. (2007). Downregulation of KLF6 is an early event in hepatocarcinogenesis, and stimulates proliferation while reducing differentiation. J. Hepatol..

[8] Narla G., DiFeo A., Yao S., Banno A., Hod E., Reeves H.L., Qiao R.F., Camacho-Vanegas O., Levine A., Kirschenbaum A. (2005). Targeted inhibition of the KLF6 splice variant, KLF6 SV1, suppresses prostate cancer cell growth and spread. Cancer Res..

[9] Reeves H.L., Narla G., Ogunbiyi O., Haq A.I., Katz A., Benzeno S., Hod E., Harpaz N., Goldberg S., Tal-Kremer S. (2004). Kruppel-like factor 6 (KLF6) is a tumor-suppressor gene frequently inactivated in colorectal cancer. Gastroenterology.

[10] Ito G., Uchiyama M., Kondo M., Mori S., Usami N., Maeda O., Kawabe T., Hasegawa Y., Shimokata K., Sekido Y. (2004). Krüppel-like factor 6 is frequently down-regulated and induces apoptosis in non-small cell lung cancer cells. Cancer Res..

[11] Cho Y.G., Kim C.J., Park C.H., Yang Y.M., Kim S.Y., Nam S.W., Lee S.H., Yoo N.J., Lee J.Y., Park W.S. (2005). Genetic alterations of the KLF6 gene in gastric cancer. Oncogene.

[12] Camacho-Vanegas O., Narla G., Teixeira M.S., DiFeo A., Misra A., Singh G., Chan A.M., Friedman S.L., Feuerstein B.G., Martignetti J.A. (2007). Functional inactivation of the KLF6 tumor suppressor gene by loss of heterozygosity and increased alternative splicing in glioblastoma. Int. J. Cancer.

[13] Chen H., Liu X., Lin J., Chen T., Feng Q., Zeng Y. (2002). Mutation analysis of KLF6 gene in human nasopharyngeal carcinomas. Ai Zheng.

[14] Bureau C., Péron J.M., Bouisson M., Danjoux M., Selves J., Bioulac-Sage P., Balabaud C., Torrisani J., Cordelier P., Buscail L. (2008). Expression of the transcription factor Klf6 in cirrhosis, macronodules, and hepatocellular carcinoma. J. Gastroenterol. Hepatol..

[15] Sirach E., Bureau C., Péron J.M., Pradayrol L., Vinel J.P., Buscail L., Cordelier P. (2007). KLF6 transcription factor protects hepatocellular carcinoma-derived cells from apoptosis. Cell Death Differ..

[16] Hanoun N., Bureau C., Diab T., Gayet O., Dusetti N., Selves J., Vinel J.-P., Buscail L., Cordelier P., Torrisani J. (2010). The SV2 variant of KLF6 is down-regulated in hepatocellular carcinoma and displays anti-proliferative and pro-apoptotic functions. J. Hepatol..

[17] Bureau C., Hanoun N., Torrisani J., Vinel J.-P., Buscail L., Cordelier P. (2009). Expression and function of Kruppel like-factors (KLF) in carcinogenesis. Curr. Genomics.

[18] Song J., Kim C.J., Cho Y.G., Kim S.Y., Nam S.W., Lee S.H., Yoo N.J., Lee J.Y., Park W.S. (2006). Genetic and epigenetic alterations of the KLF6 gene in hepatocellular carcinoma. J. Gastroenterol. Hepatol..

[19] Eberhardt W., Doller A., Akool E.-S., Pfeilschifter J. (2007). Modulation of mRNA stability as a novel therapeutic approach. Pharmacol. Ther..

[20] Elkon R., Zlotorynski E., Zeller K.I., Agami R. (2010). Major role for mRNA stability in shaping the kinetics of gene induction. BMC Genomics.

[21] Siepel A., Bejerano G., Pedersen J.S., Hinrichs A.S., Hou M., Rosenbloom K., Clawson H., Spieth J., Hillier L.W., Richards S. (2005). Evolutionarily conserved elements in vertebrate, insect, worm, and yeast genomes. Genome Res..

[22] Xie X., Lu J., Kulbokas E.J., Golub T.R., Mootha V., Lindblad-Toh K., Lander E.S., Kellis M. (2005). Systematic discovery of regulatory motifs in human promoters and 3'UTRs by comparison of several mammals. Nature.

[23] ARE site. http://rna.tbi.univie.ac.at/cgi-bin/AREsite.cgi.

[24] Tsai W.-C., Hsu S.-D., Hsu C.-S., Lai T.-C., Chen S.-J., Shen R., Huang Y., Chen H.-C., Lee C.-H., Tsai T.-F. (2012). MicroRNA-122 plays a critical role in liver homeostasis and hepatocarcinogenesis. J. Clin. Invest..

[25] Mignone F., Gissi C., Liuni S., Pesole G. (2002). Untranslated regions of mRNAs. Genome Biol..

[26] Vázquez-Chantada M., Fernández-Ramos D., Embade N., Martínez-Lopez N., Varela-Rey M., Woodhoo A., Luka Z., Wagner C., Anglim P.P., Finnell R.H. (2010). HuR/methyl-HuR and AUF1 regulate the MAT expressed during liver proliferation, differentiation, and carcinogenesis. Gastroenterology.

[27] Humeau M., Torrisani J., Cordelier P. (2013). miRNA in clinical practice: Pancreatic cancer. Clin. Biochem..

[28] Zhang X., Nie Y., Du Y., Cao J., Shen B., Li Y. (2012). MicroRNA-181a promotes gastric cancer by negatively regulating tumor suppressor KLF6. Tumor Biol..

